# Diabetic mellitus type 1 in patient with beta major thalassemia (case report)

**DOI:** 10.1186/1687-9856-2013-S1-P14

**Published:** 2013-10-03

**Authors:** Eka Intan, Fitriana Aditiawati

**Affiliations:** 1 Department of Child Health, Medical School, University of Sriwijaya/Mohammad Hoesin Hospital Palembang, Indonesia

## 

Oxidative stress in pancreatic organ may occur in children with thalassemia. It is caused by the overloaded iron store which not bound by transferrin. This condition triggers apoptosis of pancreatic beta cell so that the production of insulin decelerated progressively. Finally, it results in glucose intolerance and the patient suffers from diabetic mellitus. Some studies conclude that the risk factors of diabetic mellitus in patient with thalassemia are age, volume of blood transfusion required, high ferritin serum, history of diabetic mellitus in family, and adherence in consuming iron chelation agent. The aim of this case report to inform case of diabetic mellitus type I with beta major thalassemia in 24 year old female who has been diagnosed thalassemia since she was five months old.

Female, 24 years old, with body weight 42 kg, and height 153 cm. She admitted for routine blood transfussion because of thalassemia (previously the transfussion was done with interval of 3-4 weeks). Splenectomy was performed when she was 18 years old. So far, she has been provided therapy for desferoksamin 3x500 mg orally and intramuscular injection of deferiprone with poor compliance becaufe of side effect and availability. When she was 23 year and 9 month old, she suffered from diabetic mellitus type I and got insulin basal bolus. She also complained of having pain in left lower extremity which gradually made her very weak and couldn’t walk. Laboratory finding: blood glucose consentration: 116 g/dl, feritin: 8385 ng/mL, FT4: 0,80 ng/dl, TSH: 3,98 uIU/ml, HbA1c:10,9%, C-peptide: L< 17 pmol/L. Left cruris x-ray showed multiple tibial fracture. This patient was provided by routine blood transfussion, intramuscular injection of deferoxamine dan deferiprone 3x500 mg orally, vitamin C 2x50 mg orally, folic acid 2x1 mg orally, vitamin E 1x200 mg, calcium and vitamin D supplementation, intensive therapy of insulin basal bolus, diet DM, internal fixation. After 3 months, HbAIc level was getting improvement.

Diabetic mellitus type I in patient with thalassemia could reach optimally by doing good effort for metabolic control and by multiple approaches. Education of self monitoring on blood glucose concentration is very crucial to prevent the complication.

